# Planned adaptation and implementation of the *Community Guide* recommendations for increasing physical activity in rural community settings: A qualitative study

**DOI:** 10.3389/fpubh.2023.1032662

**Published:** 2023-03-28

**Authors:** Julia Meredith Hess, Sally M. Davis

**Affiliations:** Department of Pediatrics, Prevention Research Center, University of New Mexico, Albuquerque, NM, United States

**Keywords:** rural, implementation research, adaptation, physical activity, community-engaged research

## Abstract

**Background:**

The purpose of this paper is to report on the implementation of an evidence-based model, VIVA, which was developed to translate physical activity (PA) recommendations to rural environments and was scaled-up to 12 rural communities across New Mexico. Our longitudinal qualitative research describes processes of planned adaptation in the rural context with an exploration of inner and outer context adaptations that consider important implementation constructs including leadership, partnership and collaboration.

**Materials & methods:**

An enhanced version of the RE-AIM framework was used to formulate community-level engagement and process questions essential to implementation science. Qualitative methods, using a thematic approach that included both inductive and deductive coding with attention to processes, was used to explore adaptation at the community level. Data included semi-structured interviews with 17 community leaders at baseline and 10 at follow-up, fieldnotes, and technical assistance tracking forms. Analysis was conducted with NVivo qualitative data analysis software.

**Results:**

Analysis demonstrated how planned adaptation of the implementation model was critical to dissemination in rural communities. Understanding and adapting to local context—including geography, culture, economics—is essential for implementation. Inner context constructs, recognized as important across implementation models, including leadership, partnerships and political engagement were found to be key to implementation success. Moreover, we provide concrete examples of the range and complexity of these issues in rural communities, and how these shaped implementation uptake and success.

**Discussion:**

Studying processes of planned adaptation in rural contexts will further implementation science efforts to move evidence into practice. It is essential to incorporate planned adaptation to local, community contexts to create models which are simple to encourage adoption, are evidence-based, and are adaptable to local conditions without compromising the integrity of the evidence-based model.

## 1. Introduction

Rural health disparities have grown in the last three decades (1). Disparities in death rates, life expectancy, heart disease, diabetes, and unintentional injuries have all increased. Physical activity was identified as a top-ten rural health priority by Rural Healthy People 2020, as was nutrition, weight status, diabetes, mental health, heart disease and stroke, all of which can be addressed by physical activity (2). That these health disparities exist across large swaths of the rural U.S. underscores the need for community-based solutions that go beyond individual risk factors. Community-based and community-wide approaches are needed to address these disparities. While there is solid evidence of the role of physical activity in preventing chronic disease (3), the *how* of implementing these recommendations *in practice* in rural communities remains a complex challenge (4). This research reports on the translation and adaptation of evidence-based recommendations for increasing physical activity and their dissemination and implementation in rural communities. Learning more about how to adapt and implement successful evidence-based research in community settings is crucially important to advance efforts to address rural disparities and build on community strengths and resources to improve health and wellbeing.

In this article, we report on a multi-phased longitudinal study. Phase I included a community-university partnership to develop an evidence-based model, or prototype, which translated, disseminated, and implemented recommendations for increasing physical activity (PA) to a rural community, Cuba, New Mexico (5). The recommendations for Phase I came from *The Guide to Community Preventive Services* (*The Guide*) (6). Phase II involved scaling-up of the Village Interventions and Venues for Activity (VIVA)-Step Into Cuba model developed in Phase I to rural communities across the state of New Mexico (7). The purpose of this article is to report on the adaptation and implementation of Phase II. Our goal is to address gaps in the literature related to underreporting of how evidence-based models are adapted during the implementation phase through attention to local context in community-engaged research. All of these communities share commonalities associated with context, however, they are situated within local, regional, socioeconomic, cultural, historical, and geographic contexts that differ in important ways.

## 2. Materials and methods

### 2.1. Implementation framework

RE-AIM, an established dissemination and implementation framework, was originally developed to guide research efforts in the early stages of dissemination and implementation science to increase the speed and improve the process for bridging the gap from research to practice (8). The VIVA research team used an enhanced version of this framework to align the implementation design, process, research questions, and data collection for a scaled-up model of VIVA-Step Into Cuba. For the scale-up and implementation phase of the research, VIVA Connects, we deployed a mixed method approach to collect and analyze data guided by RE-AIM, enhanced with additional cross-cutting constructs identified by Neta et al.'s framework (9), along with Milat and Redman's success factors and barriers in scaling-up (10). The cross-cutting themes identified by Neta and colleagues include how implementation crossed multiple socio-ecological levels; a deep look at local context that goes beyond demographics and emphasizes capacity for change, leadership and communication and feedback strategies; and reporting information from multiple stakeholder perspectives (9). Thus, the enhancements do not reflect new constructs, however, more granular attention to certain constructs and how they were operationalized.

Context has long been identified as an important variable of implementation frameworks (11), however, it has also been argued that it is one of the least reported elements in research (9). Moreover, it is often limited to understanding the context of health care services settings. Because implementation science has been recognized as essential to narrow the gap between research and practice, we argue it is important to use in community settings (12) with community-engaged participatory approaches, which are shown to result in robust and valid data (13) and produce outcomes related to community goals. In research where communities comprise the implementation setting, elaboration of context is extremely important. Demarcation of inner and outer context has deepened understanding of context in implementation research (14). In community settings, outer context constructs such as legislation, policy and funding may have many points of influence. In addition, recent research on adaptation argues that examination of adaptation processes should include considerations of when and how modifications occurred, whether they are planned/unplanned, their relationship to fidelity, and reasons and goals for modification (15).

Implementation researchers have provided insight into key elements of how adaptation to local contexts occur. First, active participation of community members in all phases of the implementation (planning, implementing and monitoring) is crucial for scale-up (10). Inner context constructs such as leadership, organizational characteristics, and staffing processes can be translated to community settings. Leadership has been identified as crucial to the success of implementation efforts and is incorporated into RE-AIM and most other frameworks. Strong leadership is critical (10), and we argue that leadership should be engaged on multiple levels, from community members not engaged in local governance but who have a passion for the issue at hand, to youth and elders and others who are leaders in local institutions (e.g., health care providers, school teachers and administrators), federal, state, and other land managers, as well as elected officials. Partnerships should be examined closely and specific partnership strategies used (9). Further, local context plays an important role in what is commonly understood as the socioecological model, as can be seen in Sallis and colleagues' adapted framework, which includes consideration of intrapersonal domains, perceived environment, behavior in the context of active living, neighborhoods, workplace and school environment, and policies (16). This pragmatic approach focuses on actual, real-world settings in their broadest context.

Building on all of these insights from the implementation science literature, the research we report on suggests that applying an implementation framework that incorporates planned adaptation to local context is viable to scale-up across similar, but unique, community settings. This view of adaptation means going beyond basic community attributes, such as demographics, considering other characteristics including geography and access to public lands. An overarching question of this research can be asked: what have we learned about implementation of *The Guide* recommendations in rural contexts that may apply to other implementation research that aims increase PA?

### 2.2. Translating guidelines to a rural community: VIVA-Step Into Cuba

In Phase I, *The Guide* recommendations for increasing PA were translated through a community-academic partnership, VIVA-Step Into Cuba (2009–2014) (5). Cuba, a rural community in New Mexico served as a “beta site”, for subsequent scaling-up to similar rural communities. We therefore describe its features here as it constitutes the prototype for the adaptations in Phase II. The evidence base for VIVA comes from *The Guide*, which provides recommendations for increasing physical activity based on a review of the latest research with robust evidence of effectiveness. Much of this research, and thus many of the recommendations are grounded in urban or suburban settings (see Table 1).

**Table 1:** Examples of how planned adaptation strategies were implemented for each *Community Guide* recommendation.

**Evidence-based recommendations from *The Community Guide***	**Original research focus in metropolitan settings**	**VIVA-Step Into Cuba: phase I translation for rural beta site**	**VIVA Connects: phase II adaptation for scale-up across rural areas of state**
Community-wide campaign	• Involve many community sectors • Involve visible, broad-based, multi-component strategies	• Used variety of communication channels, including project website (www.stepintocuba.org), local media (newspaper, posters, signs radio) • Held and promoted Community events such as “Full Moon Hike”, school class nature walks • Created kiosks and signs in community	• Developed and promoted walking groups led by community members, for example “Walk with a Doc”, “Walk with a Birder”, “Walk with a dog” (local shelter animals) • Local media: radio; flyers in utility bills; trail signs; newspaper
Create or enhance access to places for physical activity with informational outreach	• Focus on urban settings [e.g., green spaces, parks. exercise facilities (e.g., health clubs, YMCA)]	• Created, enhanced, and promoted 20 miles of trails in 7 locations • Enhanced local park: planted trees, wildflowers, shrubs, installed benches; produced walking guide and mobile app with trail information; engaged volunteers • Re-routed trail to enhance connectivity with Continental Divide Trail • Created maps and walking guide and mobile app with trail information • Created trail and town signage	• Promoted walking route with information on its cultural history in the community • Planned new trail to create connectivity between State Park and town • Equestrian, mountain biking and walking trails to contribute to economic development • Improved and promoted trail around DUI memorial park • Walkability workshop results adopted for city planning • Expanded mobile app to include VIVA Action Communities
Individually adapted programs	• Focus on behavior change through goal setting, skill building and self-monitoring of goals; building social support for new behavioral patterns	• Promoted walking through physical activity prescriptions from local health care providers. This was not effective.	• Evidence-base did not support this option; so de-emphasized
Social support for physical activity	• Individual enrollment in physical activity with social support component (in person or virtual check-ins; group component)	• Recruited walking champions who led walking groups for specific populations (e.g., seniors; employees; elementary and middle school students) • Developed and promoted community events (hikes, walks, runs) • Promoted benefits of walking as widely accessible and effective exercise	• Developed and promoted walking groups (see above) • Developed and promoted community events (hikes, walks, runs)
Street-scale design & land-use policies	• Combined efforts or urban planners, architects, engineers, developers and public health professionals to change physical environment in small geographic area. • Improved lighting, crossing safety, traffic calming, landscaping	• Conducted an HIA for highway improvements • Provided technical assistance with applications for creation and improvement of sidewalks • Provided recommendations for fairgrounds development • Completed memorandum of understanding with local school to allow community use of cross-country trail	• Completed walkability assessments for sidewalks, cross- walks and pedestrian safety • Park trail improvements • Connectivity between local and state parks
Other	• Not a *Community Guide* recommendation, but our concern with sustainability and community needs prompted us to address in our TA.	• Provided technical assistance to leverage funding for projects	• Provided technical assistance to leverage funding • Developed partnership with New Mexico Department of Health to provide mini-grants to Action Communities

Recommendations related to increasing access to places to be physically active, community-wide campaigns, individually adapted behavior change programs, social support, and the built environment were translated to the rural context simultaneously and on multiple levels of the socioecological model through the creation of a logic model which guided the project through its phases (5). Community-wide campaign guidance included involving many community sectors, including highly visible, broad-based, multicomponent strategies (e.g., social support, risk factor screening or health education). In Phase I, this recommendation was adapted to the creation of a website, the production of walking guides to promote places to be physically active, which eventually led to the creation of web-based and mobile phone application with trail maps and information. Walking was also promoted in the local newspaper, and through outdoor kiosks promoting specific trails and signs encouraging people to walk for health or convenience at the post office, clinic and credit union. Additional strategies included a walking champion who led walking groups for seniors, employees and students. For more on the results of this phase of the study see (17).

### 2.3. Widespread dissemination, scale-up and implementation across rural New Mexico: VIVA Connects

The second phase of research, 2014–2019, involved scale-up and implementation of the beta site prototype to other rural communities across New Mexico to see if it could be successful in communities with similar attributes such as being rural and under-resourced, but each with unique geography, political climate, natural resources, culture, and history. One hundred sixty-five communities with a population between 500 and 12,000 were originally identified using U.S. Census county-level data for New Mexico. We recruited participating communities from this list of eligible communities by distributing a form to those with which we had previous relations, often at conferences, through the health department and previous contacts, a website, videos, factsheets and a listserv. Of those 165 communities, a total of 31 communities chose to be included in the network by completing a VIVA Connects Action Community Intake form. Each of the 31 communities were invited to submit requests for technical assistance (TA) to implement activities to increase PA in their communities. Leaders from the community were able to request TA related to increasing PA in their communities. These TA requests were categorized using a form to indicate which *Community Guide* recommendation was represented to ensure they fit into the evidence base. The form used a “stoplight” format with green, yellow and red sections to categorize the TA requests, indicating whether or not the TA could be completed immediately (green), were achievable, but would take some time (yellow), or were not within our scope or were not considered evidence-based (red). Communities that (1) demonstrated active interest in implementing evidence-based recommendations to increase PA, (2) identified one or more community champions to assess needs and were involved in coalition-building around PA, and (3) requested TA on at least 2 occasions, were invited to be VIVA Connects Action Communities. Action Communities (*n* = 12) were then included in the qualitative arm of the research study.

Following *Diffusion of Innovations*, we wanted to highlight the importance of intermediary actors, or opinion leaders and change agents. Therefore, we refer to the leaders identified in these communities as “champions” in an effort to broaden the concept beyond political or other more traditional leaders (18). Thus, in this context, “champions” are people who took on a leadership role in a community directly related to increasing access or enhancing places to be physically active and who expressed interest in participating in the network of all 31 communities, VIVA Connects. The network was important as it allowed Action Communities and others who were interested, but not yet requesting TA, to share resources, ideas, and successes with each other, to share insights about successes overcoming challenges often particular to rural communities. Sharing was facilitated by participation in a listserv, learning modules accessed through a website, and the VIVA Connects website.

### 2.4. Data collection

The first author conducted semi-structured interviews with 17 champions from 12 Action Communities in a baseline interview after joining the study. She has a Ph.D. in cultural anthropology and served as the lead on the qualitative strand of this study and had over 20 years' experience conducting qualitative research. She had no relationship with study participants prior to the research being conducted. She conducted some interviews with individuals and others with groups of more than one champion. The interview guide remained consistent for interviews of individuals and groups. Because of the broad conceptualization of leadership from different sectors, champions represented stakeholders ranging from department of health employees, members of local health councils, state park rangers, city planners, and rural extension agents. Fifteen were female; we did not collect race/ethnicity, age or other demographic information. Champions were contacted *via* phone or email and invited to participate. Interview questions were open-ended and covered multiple domains related to community goals around physical activity, based on *The Guide's* evidence-based recommendations, and community adaptations or extensions of the VIVA model according to local context. We also asked about key factors included in the enhanced RE-AIM framework, specifically community readiness, coalition-building, partnerships, political leadership, and local context. Interview questions were pilot tested internally with members of the research team. Follow-up interviews were conducted with 10 participants from eight action communities after at least 1 year of participation in VIVA Connects. Many of the communities had experienced leadership changes and new people were included in the interview in addition to the initial interviewee (*n* = 4) or were interviewed in their stead (*n* = 1). Four action communities experienced change in leadership and the originally identified champions were not available for follow-up interviews, and no new champions could be identified and interviewed in their place. Topics included progress on goals, reflections on how previously explored domains (e.g., leadership and partnerships) affected progress in improving access to places for PA. Sustainability was also discussed. Interviews were conducted in person or *via* telephone with champions. Interviews, whether in person or over the telephone, were conducted in private offices or conference rooms and ranged from 30 min to 1.5 h, averaging 56 min. Interviews were not recorded, however, responses were transcribed by the interviewer during the course of the interview. These transcripts were very close to verbatim, omitting filler words and false starts, but attempting to capture participant speech as accurately as possible. Written consent was obtained and the research was approved by the university's institutional review board. Transcripts were sent to interviewees to allow for correction and/or additional elaboration. Data saturation was not a goal as our research design included interviews with all community champions.

In addition to interviews, VIVA Connects staff provided TA in person, by email, or by telephone, and through web-based learning modules available to the network of participating communities. Site visits included the coordination and leading of community-level assessments of places to be physically active. TA GO forms were completed by staff to track and describe TA provided, including which *Community Guide* recommendation was followed in each case. Fieldnotes of these site visits and each contact when staff interacted directly with the community were collected, imported into NVivo, and coded with the same coding tree and were thus included in our analytic memo writing process.

### 2.5. Data analysis

Data were analyzed using a thematic approach. We used a two-phased coding cycle approach that combined inductive and deductive analysis. Interview transcripts, fieldnotes and TA GO forms were anonymized, formatted and imported into NVivo 11 qualitative data analysis software (19). In the first coding cycle, interviews, observations, meeting notes and other text were coded using primarily descriptive codes, hewing closely to participant language. We also used process and values-coding techniques, resulting in a coding tree developed by the first author (20). Codes were created both deductively and inductively based on questions derived from the enhanced RE-AIM framework (e.g., adaptation, local context, partnerships, leadership and coalition building) and emerging themes. Others were related to the evidence-based intervention strategies found in *The Guide* (e.g., increasing access to places to be physically active, community-wide campaigns). The first author also trained two team members in qualitative coding (one medical student and one intern from the Centers for Disease Control) who conducted first cycle coding. The largely descriptive coding tree resulted in high levels of agreement (>0.75 Kappa co-efficient) when conducting inter-coder reliability checks. Data collected at these codes were then analyzed using second cycle, focused coding techniques (21). Memos were created on each of these thematic constructs (e.g., adaptation; partnerships) and evidence-based recommendation categories (e.g., increasing access to places). The process of memo writing includes reviewing all data associated with a code (or collection of codes) and organizing it in sub-categories, looking for patterns, anomalies, and suggesting other themes or coding intersections to explore. This is where the majority of interpretation and analysis occurred. In addition, queries were used to analyze facilitators and barriers related to the inner context constructs (e.g., coalitions, leadership, and partnerships) for each Action Community and how these changed over time (e.g., from the first to the second interview). Each Action Community became a “case”, and we reviewed all associated data chronologically to assess change over time. Additional memos were created to track and analyze important phenomena that affected the research and community implementation, for example, frequent turnover in leadership made it difficult to re-interview champions from the baseline interview and in some communities, thus we created a memo “Turnover, Leadership Issue”. We also created visual matrices based on these data to examine inner and outer constructs across communities, as well as “milestones” of implementation success (e.g., creation of maps, walkability assessments, creation of walking guides) to better understand facilitators and barriers regarding these constructs and milestones. These memos and matrices form the basis for the results presented in this article.

## 3. Results

Baseline interviews with community champions provided local context information that was used for the planned adaptation process that began as soon as possible after communities joined VIVA Connects. Adaptation of the Phase I prototype was led by community champions as they gained knowledge of the evidence base, shared local context elements with the research team, received TA, and participated in a network of other rural communities trying to achieve similar goals in their own communities.

### 3.1. Outer context adaptations

Collecting data about context and encouraging implementation in community settings with approaches that consider local geography, culture, and economics, underscores the planned adaptation features of our modified implementation model (15). For our purposes, these aspects—geography, culture, economics, and land use—consist of outer context elements (22) in community settings. Interview participants mentioned many strengths of rural contexts that communities can build upon to increase PA. In addition, identification of strengths confirmed important aspects of adaptation to rural context noted in Phase I of the project, the translation of evidence-based recommendations for a rural community in the beta site.

First, among common rural strengths is proximity and access to public lands. U.S. National Forest, Bureau of Land Management, National Park Service, state, county, tribal and other publicly funded and managed lands provide outdoor settings for physical activity. Places to be physically active in town, such as parks, if present, are also important, similar to urban settings.

In Phase II, local adaptations of this recommendation took on various forms, principally highlighting the way local, place-related historical and cultural information can be incorporated into efforts to increase access. For example, the VIVA Connects Action Community coalition in Tularosa had the goal of improving accessibility on a commonly used walking route that followed historic *acequias*, or Spanish colonial irrigation ditches that also served to link sacred cultural history to current practices. Ideas were to create signage that provided walkers information about the area's history, including QR codes to access more text, audio, and visual material related to the walking trail.

Another example is Moriarty, a rural community that had identified few places in which to be physically active and had no close proximity to public lands. In response to the prevalence of deaths caused by driving under the influence of alcohol, a state-wide memorial had been built in the community, consisting of a field of markers designed to look like gravestones to represent the last 5 years of state-wide fatalities related to driving while intoxicated. Members of the county-wide coalition recognized that this accessible, public space could serve as a place for a walking trail. Champions made plans to develop and grade a walking trail around the perimeter of the memorial. Walking the trail could serve as an act of remembrance and provide a safe space for community members who wish to walk on a regular basis. A city in the southern part of the state, Silver City, created a multi-group coalition and capitalized on proximity to the Continental Divide Trail (CDT) and being designated a CDT Gateway Community. The coalition also worked with local government to purchase inactive mining sites to create trails, which were promoted through a community-wide campaign and signage. In these ways, pre-existing land use can be enhanced to create safe, accessible walking trails for communities.

Large geographic areas typical of rural contexts made the focus on connectivity important. For example, Ramblin' Round Raton, a VIVA Connects Action coalition in Raton, created connectivity between a town park and Sugarite Canyon State Park approximately 6 miles away, through a trail to improve pedestrian access and usage of both sites. Another aspect of this recommendation includes a focus on walkability, which is important in rural and urban contexts alike. Improving sidewalks, creating crosswalks, and decreasing motorized traffic speeds are critical for improving walkability in rural areas, however, whereas in urban settings these projects make up a small percentage of municipal budgets, they are often cost prohibitive in rural communities without leveraging funding and expertise from multiple sources. Therefore, VIVA Connects became a source of technical assistance to access this funding to make these important improvements.

Working under a broad vision to improve the health of the community leaves room for various motivations, including economic development. Therefore, VIVA Connects adaptations focused not solely on walking, but on increasing other kinds of non-motorized traffic, for example, making trails accessible for equestrians as well as mountain bikers. Edgewood, population around 4,000, has worked to enhance multi-use trails for walkers, equestrians, and mountain bikers in conjunction with economic development initiatives supported by the Chamber of Commerce, the local parks and recreation department, and a hiking group. In addition, many of these communities were interested in connectivity—between trail systems, connecting trails to parks, and often increasing connectivity between schools, clinics, and other places to make it safer and easier to walk or bike through town. For example, in Taos, a champion stated “and if we can get agreement with town to connect to Fred Baca park. As a part of the town's planning process, she found some cool connections, and found a potential site for dog park on a town property a couple of parcels down. That would be great a connection site”. In Silver City, the champion emphasized the importance of connecting the CDT to town: “CDT, having Silver City truly connected, a gateway community. Even though there is not a trail connecting, but that's what people want. Largely for economic development, to help thru-hikers have access that's not a highway, it's out of the way, off the trail”. In Edgewood, the champion spoke of getting a trail connected to another trail near a concentrated population so they can get access to “this other set of trails. … Connectivity is key”.

Implementation models that include planned adaptation strategies tied to evidence-based recommendations adapted to local conditions serve to highlight the ways technical assistance and networking between communities promote successful strategies to improve individual communities. Moreover, enacting the creation of a network of communities to share and build on evidence-based strategies adds to the overall success of each community and the implementation as a whole. Table 1 shows how evidence-based recommendations from *The Guide* were translated in the beta site in Phase I and then further adapted in the scale-up in Phase II.

### 3.2. Inner context adaptations

Inner context includes leadership, partnerships, and collaboration related to conducting implementation in the community setting (22). We identified commonalities related to the inner context across all the rural communities in this study.

#### 3.2.1. Leadership

With respect to leadership at the community level, common barriers mentioned by champions included distrust of outsiders, “turf guarding” (defending one's area of influence and being resistant to working with others), programmatic silos, and lack of knowledge about how other rural communities addressed these issues. Some mentioned that community members and political leaders can be averse to change. Leaders said that highlighting the preventive aspect of PA in relation to community-wide health concerns is a hard sell given the tight funding environment. Leaders in rural communities endeavored to create coalitions of people with diverse roles and interests united by the common motivation to improve the health of their communities. In addition, the turnover of community champions themselves was indicative of unstable funding or other challenges that led champions to seek employment elsewhere. In rural contexts, adaptations include the necessity of including a variety of land managers from different agencies (e.g., Bureau of Land Management, U.S. Forest Service, and State Parks) as well as planners, health providers and others. However, we saw that if a coalition was not well-established, leadership changes or vacuums in leadership led to delays or perhaps even abandonment of previously set goals in the community around increasing access to places to be physically active.

#### 3.2.2. Partnership and collaboration

Viewing leadership as broader than local political leaders is important in every context but is critical in rural communities where population is low, and leadership in multiple sectors must be cultivated. Building coalitions with a broad vision—improving community health—encourages bringing in and cultivating many different kinds of leaders with experience in the community.

Moreover, leaders spoke about including partners with specific areas of expertise, who have critical knowledge about how to maneuver within complex systems, but also have links to other experts in associated realms who can help accomplish goals. Thus, diverse coalitions made of partners with different areas of expertise, age, gender, and ethnicity all contribute to diversity. A community champion reported the benefits of a diverse coalition:

Since I started attending, founders and elders were like, “Heck yeah, I am retired, let's build a trail.”... In rural communities, you realize there are a lot of people you have to ask for permission. In the last meeting, we had US Forest Service, county commissioners, a county mapping and planning person, the National Park Service, [a local conservation organization], trying to help with wilderness area, and [mine company representatives]. They have mines all over the place.... It's not a matter of just asking permission, it's getting people at the table: DOT [Department of Transportation], council of governments. So when so-and-so says they are not going to let that happen, we can say, “Hey, so-and-so, how can we make that happen?” We can have more progressive dialogue. If people are investing time they are more committed [Participant 0043].

The champion is also demonstrating the kind of expertise and commitment needed to navigate the complexity of the local context, along with knowledge of how to best leverage leadership to achieve results.

Adaptations for the rural context included developing a community-wide vision built on wider goals than those related to physical activity (e.g., improving community health), forming a diverse coalition with leaders from different sectors, having people with local roots as leaders, and providing a context or mechanism for elders or people with seniority, to pass knowledge down to younger people.

Technical assistance requests were categorized according to *The Guide* recommendations. It was notable that many requests fell into the “other” category, specifically funding. Federal, state, and other governmental sources of funding to make places more walkable are tailored to the resources and capacity of larger communities, including full-time staff dedicated to grant writing and management was often mentioned as a challenge. In a group interview with several champions from one community, they discussed this issue. One community champion said,

My frustration with the planning process, everyone is up here [motions with hands like a ceiling or line above his head], we are down here [motioning near the floor]. When you go to RTPO [Rural Transportation Planning Organization], DOT has grants available to communities, $75,000 is smallest one, with a 20% match, they want bigger things, that's not where we are. [Someone from the planning agency] asks, “Can't you come up with 10% of $500,000?” “No! We can't!” You want us to invest in walking, but we don't have this in our budget. It's a hard sell [Participant 029].

Economic challenges included PA being low on the list of priorities in communities affected by the opioid crisis, lack of jobs, and other urgent and systemic issues. Funding challenges underscore the interrelated inner and outer context dimensions and how they impact rural communities in common ways, revealing needed structural and policy changes to address rural disparities.

Our qualitative data led us to suggest that some Action Communities were not as successful in their efforts to increase physical activity through applying *The Guide* recommendations even with planned adaptation around outer context conditions. Using queries and matrices to compare qualitative data from our Action Communities, the interpretation of our research team was that inner context constructs were vitally important. Those who had more developed, diverse coalitions, local leadership, and diverse partnerships with expertise to address specific barriers created by context, were more successful in creating or enhancing places to be physically active in the community.

## 4. Discussion

Rural health disparities present an urgent public health problem that can be addressed at the community level. Research in urban contexts has provided strategies to increase access to places to be physically active, but these must be translated to rural contexts. Rather than using a deficit perspective for rural communities, which focuses on declining physical activity rates and the rise in chronic conditions, our research is focused on community strengths. Common strengths of rural communities include proximity to public lands, which considered together with socioeconomic and cultural contexts, can be built upon to increase physical activity and thus, decrease health disparities.

As implementation science has developed more consensus about common constructs, there are specific processes that occur within implementation that illustrate the need for a pragmatic approach. This is especially important in community-engaged research, where understanding and adapting to local context is an essential part of implementation. Interventions that have demonstrated viability can be scaled-up to similar settings, however, attention to local context is critical for success. Thus, adaptation is a critical feature of scaling-up evidence-based interventions. Researchers have noted that a lack of attention to adaptation may be a legacy of empirical models that have relied heavily on conducting science in controlled conditions, which is problematic in real world practice settings (23). The tension between adaptation and fidelity may have hampered the willingness to fully explore the need for adaptation and adaptation processes. In community-engaged research, adaptation to local context is not only important, but essential to implementation success. Further, its study should be included as part of the research activities.

Studying adaptation processes in community-based settings underscores the need for a broad understanding of context that not only goes beyond demographics, but considers geography, culture, politics and that these are constantly in flux. This complexity and changeability make it critical to include planned adaptation strategies as part of the implementation approach. Flexible models that include adaptation to local context as part of the implementation process and provide parameters for guiding adaptation are critical to improving chances of adoption and positive outcomes related to the intervention.

Conducting translation of evidence-based guidelines for increasing physical activity in a specific rural community (Cuba, New Mexico) did not provide the team with a one-size-fits-all model for scaling-up to other rural communities. It did, however, provide the team with an idea of how to incorporate planned adaptation strategies into the model for implementation in other communities with commonalities across geographic, cultural, political and economic configurations.

Planned adaptation in the VIVA model was directed at the outer context, or the geography, land use practices, economic and other context-specific features of the rural communities where the implementation occurred. However, our results showed the importance of inner context, which addresses leadership and other aspects of the organization or coalition doing the work. Future efforts will be sure to attend to adaptations of inner context, including, for example, how to build coalitions and partnerships critical to rural contexts. In addition, it is important for coalitions to enact these practices related inner context elements during the implementation itself, for example, participating in partnership networks to share ideas, successes, and brainstorm how to overcome obstacles to their efforts that are often common across settings.

In addition, although *The Guide* does not include providing technical assistance regarding leveraging funding as a recommendation, community needs made this a priority in VIVA Connects. Funding challenges underscore the interrelated inner and outer context dimensions and how they often impact rural communities, revealing needed structural and policy changes to address rural disparities.

This planned adaptation process necessarily combines both research and implementation efforts: namely, understanding and assessing local context including geography, cultural, political and economic landscape, historical patterns, and in-depth interviews with community champions to understand local manifestations of cross-cutting elements that have been identified as essential to successful implementation efforts: (e.g., geography, culture, land use patterns, leadership, partnerships, and political engagement). This process, while framed as data collection, also provides a guide for which factors local champions consider as potentially important to their efforts to create change around PA in their communities. Follow-up interviews can also aid in this purpose, as they can track change over time to evaluate the outcomes of intensive TA efforts, and help researchers, implementers and community members understand how local context is contributing to needed adaptations. The experience of the VIVA team, although conceptualized as research, holds important pragmatic lessons for communities interested in efforts to increase physical activity, including health care providers, policy makers and other implementers. We suggest that adaptation to context may contribute to sustainability of efforts over time, however, this is an area for future research.

### 4.1. Limitations and future directions

Although implementation costs have been identified as important to include in implementation science research, we had not planned on providing TA for communities around funding. However, this is clearly an issue for community leaders and the rural context indicates unique dimensions of the problem and thus, much of our TA, focus of content shared in the VIVA Connects network, and eventual successes were related to accessing and leveraging funding.

Qualitative methods are essential to study how processes unfold, especially in community settings with multiple contexts and levels. A deep focus on local context can limit generalizability, however, rich descriptions that show how adaptation is accomplished and its effects, can be widely applied. An implementation framework that attended to adaptation processes that relate to both outer (specifically focused on attributes of the research setting) and inner constructs, (in particular, leadership, partnerships, and collaborative processes) would provide an excellent foundation for future studies. Qualitative analysis pointed to some important relationships between the robustness of inner and outer context constructs and how successful communities were in reaching milestones, however, a more robust mixed method approach would be needed to provide more solid evidence of the association. Mixed methods could provide a robust quantitative component to analyze to what extent these constructs contributed to success. Adaptation measures that include inner and outer constructs are essential.

## 5. Conclusion

Implementation science has relevancy beyond institutional settings and has important applications in rural community settings. Implementation science has identified a core of common constructs that are important to address when implementing research and programs. These apply to community settings. Our research demonstrates the importance of implementation that is both built on evidence related to the desired outcomes (e.g., increasing physical activity) and implementation science (e.g., using established frameworks to guide research questions and implementation activities). It is essential to incorporate planned adaptation to local contexts and be mindful to how these processes encourage adoption, are evidence-based, and yet are adaptable to local conditions without compromising fidelity.

## Data availability statement

The original contributions presented in the study are included in the article/supplementary files, further inquiries can be directed to the corresponding author.

## Ethics statement

The studies involving human participants were reviewed and approved by University of New Mexico Health Sciences Human Research Protections Program. The patients/participants provided their written informed consent to participate in this study.

## Author contributions

SD designed the study and contributed substantially to paper organization, content, and editing. JH integrated RE-AIM framework with qualitative questions, conducted the analysis, and drafted the paper. JH and SD read and approved the final manuscript. Both authors contributed to the article and approved the submitted version.
